# Stress Hyperglycemia in Patients With Acute Ischemic Stroke Due to Large Vessel Occlusion Undergoing Mechanical Thrombectomy

**DOI:** 10.3389/fneur.2021.725002

**Published:** 2021-09-29

**Authors:** Giovanni Merlino, Sara Pez, Gian Luigi Gigli, Massimo Sponza, Simone Lorenzut, Andrea Surcinelli, Carmelo Smeralda, Mariarosaria Valente

**Affiliations:** ^1^Stroke Unit, Department of Neuroscience, Udine University Hospital, Udine, Italy; ^2^Clinical Neurology, Udine University Hospital, Udine, Italy; ^3^Dipartimento di Area Medica (DAME), University of Udine, Udine, Italy; ^4^Division of Vascular and Interventional Radiology, Udine University Hospital, Udine, Italy

**Keywords:** stress hyperglycemia, GAR index, acute ischemic stroke, outcome, mechanical thrombectomy

## Abstract

Stress hyperglycemia may impair outcomes in patients with acute ischemic stroke (AIS) undergoing mechanical thrombectomy (MT). The glucose-to-glycated hemoglobin ratio (GAR) was used to measure stress hyperglycemia. Data from our database of consecutive patients admitted to the Udine University Hospital with AIS who were treated with MT between January 2015 and December 2020 were retrospectively analyzed. We included 204 patients in the study and stratified them into four groups according to the quartiles of GAR (Q1–Q4). The higher the GAR index, the more severe the stress hyperglycemia was considered. Patients with more severe stress hyperglycemia showed a higher prevalence of 3-month poor outcome (Q1, 53.1%; Q2, 40.4%; Q3, 63.5%; Q4, 82.4%; *p* = 0.001), 3-month mortality (Q1, 14.3%; Q2, 11.5%; Q3, 15.4%; Q4, 31.4%; *p* = 0.001), and symptomatic intracranial hemorrhage (Q1, 2%; Q2, 7.7%; Q3, 7.7%; Q4, 25.4%; *p* = 0.001). After controlling for several confounders, severe stress hyperglycemia remained a significant predictor of 3-month poor outcome (OR 4.52, 95% CI 1.4–14.62, *p* = 0.012), 3-month mortality (OR 3.55, 95% CI 1.02–12.29, *p* = 0.046), and symptomatic intracranial hemorrhage (OR 6.89, 95% CI 1.87–25.36, *p* = 0.004). In summary, stress hyperglycemia, as measured by the GAR index, is associated with a detrimental effect in patients with AIS undergoing MT.

## Introduction

Mechanical thrombectomy (MT) is the “gold standard treatment” for patients with acute ischemic stroke (AIS) due to large vessel occlusion (LVO) (1). In contrast to randomized controlled trials that reported a prevalence of 3-month good outcomes after MT as high as 71% (2–6), lower rates of patients with functional independence, ranging between 34 and 39%, were reported by observational registries (7–9).

Glycemic status should be considered one of the most important modifiable predictors of poor outcomes in patients undergoing MT. Several mechanisms, such as alteration of the blood barrier permeability (10), exacerbation of the thromboinflammatory cascade (11), acidosis (12), and increased oxidative stress response (13), explain the poor outcome in AIS patients with hyperglycemia. Hyperglycemia at admission has been associated with a decreased likelihood of good outcomes in patients with AIS undergoing MT (14–17). However, we recently demonstrated that persistent hyperglycemia—that is, hyperglycemia at admission and at 24 h post-admission—was a better predictor of poor outcome than baseline hyperglycemia in AIS subjects affected by LVO and treated with MT (18). Persistent hyperglycemia might impair outcomes as a marker of either diabetes mellitus or activation of the hypothalamic–pituitary–adrenal axis, that is, stress hyperglycemia. Although diabetes is a widely recognized predictor of stroke (19), little data are available on the possible detrimental effects of stress hyperglycemia in AIS patients undergoing recanalization therapy (20–22). In particular, only two recent Chinese studies reported that stress hyperglycemia was a strong predictor of poor clinical outcomes and mortality after MT (21, 22). To date, similar data in Caucasian patients are lacking.

Recently, the glucose-to-glycated hemoglobin (HbA1c) ratio (GAR) has been developed to assess stress hyperglycemia (23). In particular, Su et al. (23) performed a retrospective observational study to investigate if the acute elevation of plasma glucose among patients visiting the emergency department was associated with poor clinical outcomes. In order to distinguish between the stress-related hyperglycemia, due to acute illness, and the patient's premorbid glycemic condition, the authors introduced a new index, the GAR. The GAR index was calculated as the plasma glucose concentration divided by HbA1c (23). In contrast to HbA1c, which identifies the baseline average glucose status over the past 3 months, the GAR index measures the presence of acute elevation in plasma glucose, that is, stress hyperglycemia, in comparison with background plasma glucose levels.

In the present study, we investigated the role of stress hyperglycemia, measured by the GAR index, as a predictor of poor outcome in AIS patients treated with MT.

## Materials and Methods

### Study Participants

Data from our database of consecutive patients admitted to the Udine University Hospital with AIS due to LVO who were treated with MT between January 2015 and December 2020 were analyzed. The patients were followed up for 3 months. The eligibility criteria for MT were as follows: (i) presence of LVO in the anterior or posterior circulation as revealed by CT angiography (CTA), (ii) onset of symptoms within 6 h, and (iii) Alberta Stroke Program Early CT Score (ASPECTS) >6 on a direct CT scan (24). In contrast, patients with a life expectancy of <6 months, severe medical conditions with signs of organ failure, and platelet count <55,000/mmc are not treated with MT at our center. According to the international guidelines, alteplase was used to treat AIS patients showing onset of symptoms within 4.5 h (1). A follow-up CT scan was performed approximately 24 h after recanalization therapy or sooner if clinical deterioration was observed. Written informed consent was obtained from all patients or their representatives. The study conformed to the Declaration of Helsinki of the World Medical Association and was approved by the local ethics committee, *Comitato Etico Unico Regionale* (Ref. No. CEUR-2020-Os-173).

### Data Collection

We collected the following information: age, sex, laboratory findings, systolic blood pressure at admission, previous pharmacological treatment, and vascular risk factors, including previous transient ischemic attack or stroke, cardiovascular disease, atrial fibrillation, hypertension, diabetes mellitus, hypercholesterolemia, and active tobacco use. A history of diabetes mellitus that had been confirmed in medical records and/or the use of insulin/oral hypoglycemic agents were considered for defining diabetes. The ASPECTS score was used for grading early ischemic changes within the middle cerebral artery (MCA) territory on a native CT scan (24).

### Clinical Assessment

According to the Trial of ORG 10172 in Acute Stroke Treatment (TOAST) criteria, ischemic strokes are classified into different subtypes based on etiology (25). Stroke severity was quantified at admission and discharge using the National Institutes of Health Stroke Scale (NIHSS) score. Major neurological improvement was defined as an improvement of ≥8 points on the NIHSS score from baseline or an NIHSS score of 0 or 1 at discharge. The degree of previous functional disability was calculated at admission, based on pre-stroke disability, and 3 months after stroke using the modified Rankin Scale (mRS). The mRS score after discharge was recorded during the patients' routine clinical visits or through telephone interviews with patients or their immediate caregivers. We dichotomized mRS into favorable outcomes (0–2) and poor outcomes (3–6). The European Cooperative Acute Stroke Study (ECASS) definition of parenchymal hematoma types 1 and 2 was adopted to identify intracranial hemorrhage (ICH) (26), whereas the presence of symptomatic intracranial hemorrhage (SICH) was based on the ECASS III protocol (27).

### Thrombectomy Procedure

The following information was collected: site of the cerebral artery occlusion, type of device used for MT procedure, presence or absence of secondary embolization, time from onset of symptoms to MT, time from hospital arrival to groin puncture (door-to-groin time), procedure duration, and successful recanalization rate, defined as thrombolysis in cerebral infarction (TICI) score of 2b−3. In addition, if patients received alteplase; we collected information on the time from onset of symptoms and hospital arrival to alteplase administration (door-to-needle time).

### Assessment of Stress Hyperglycemia

For laboratory tests, including fasting plasma glucose and HbA1c, venous blood samples were drawn within 24 h after hospitalization, during the morning hours (range: 06:00–08:00) after an overnight fast (at least 12 h). Stress hyperglycemia was estimated using the GAR index. The GAR index was calculated as fasting plasma glucose (mg/dl)/HbA1c (%). We stratified patients into four groups according to quartiles of GAR (Q1–Q4) for further comparisons. The higher the GAR index, the more severe the stress hyperglycemia was considered.

### Outcome Measures

The following endpoints were analyzed: 3-month poor outcome, no major neurological improvement at discharge, 3-month all-cause mortality, in-hospital all-cause mortality, presence of SICH, and presence of ICH. All outcome measures were collected as part of routine clinical practice in patients affected by cerebrovascular events.

### Statistical Analysis

Data are displayed in tables as mean and standard deviation unless otherwise specified.

Statistical comparisons were performed using the chi-square test or Fisher's exact test, when appropriate, for categorical variables. One-way analysis of variance for normally distributed continuous variables and the Kruskal–Wallis test for non-normally distributed continuous variables and ordinal variables were used. The Bonferroni–Dunn *post-hoc* test was used for *post-hoc* analysis. The Kolmogorov–Smirnov test with Lilliefors significant correction was performed to test the normality of the variables.

Multivariable logistic regression was performed to confirm the role of stress hyperglycemia, represented by GAR quartiles, as independent predictors of outcome in AIS patients undergoing MT. We used the lowest GAR quartile as the reference category. The regression model was adjusted for age, history of diabetes, ASPECTS score, baseline NIHSS score, pre-stroke mRS, time from symptom onset to MT, door-to-groin time, and procedure duration. We decided to add systolic blood pressure to other confounders in the analysis to evaluate the association between stress hyperglycemia and hemorrhagic transformation (i.e., SICH, ICH).

All probability values were two-tailed. Statistical significance was set at *p* < 0.05. Statistical analysis was carried out using IBM SPSS Statistics for Windows, version 22.0 (IBM Corp., Armonk, NY, USA).

## Results

### Baseline Characteristics

**Figure 1:**
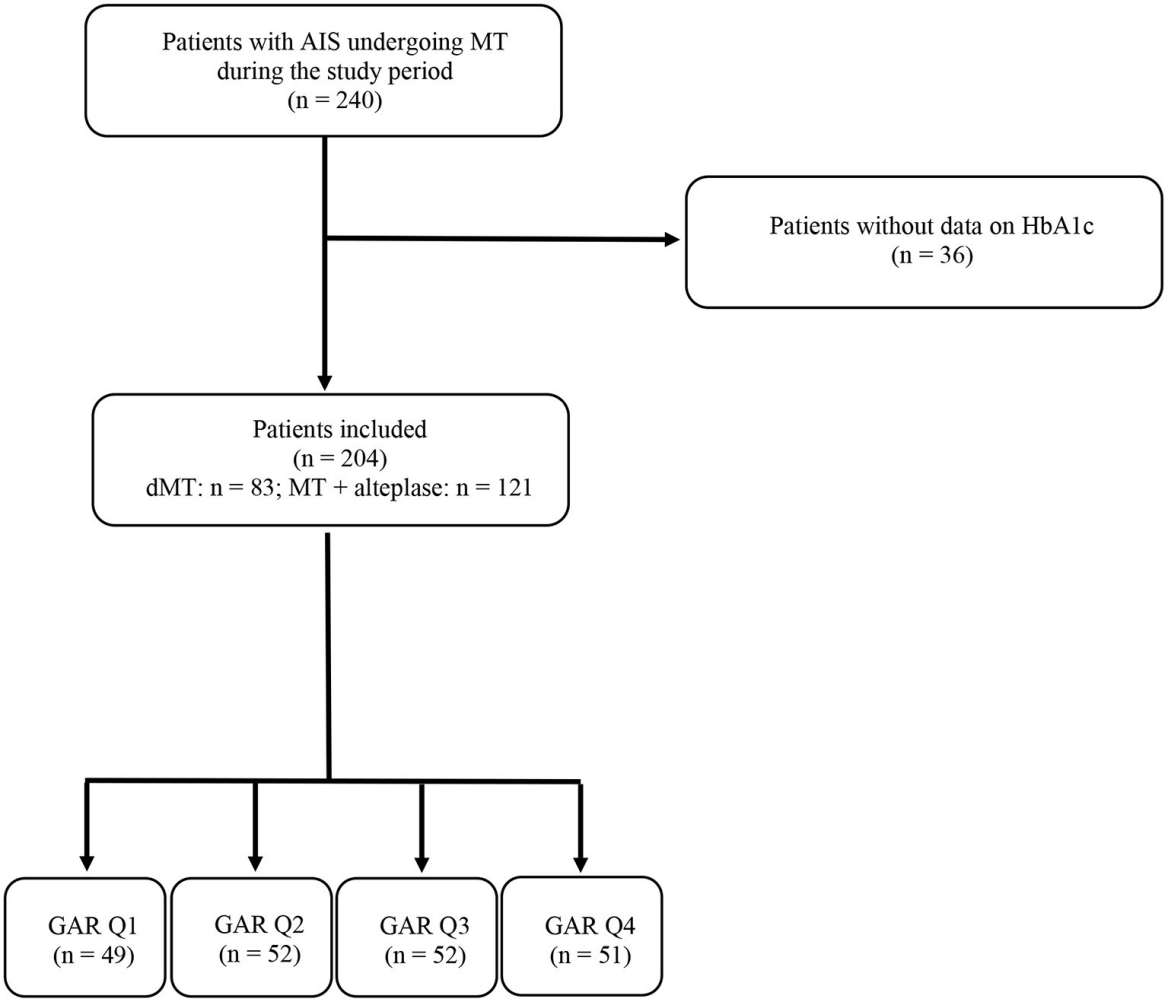
Flow diagram of the study. AIS, acute ischemic stroke; MT, mechanical thrombectomy; HbA1c, glycated hemoglobin; dMT, direct mechanical thrombectomy; GAR Q1, first glucose-to-glycated hemoglobin ratio quartile; GAR Q2, second glucose-to-glycated hemoglobin ratio quartile; GAR Q3, third glucose-to-glycated hemoglobin ratio quartile; GAR Q4, fourth glucose-to-glycated hemoglobin ratio quartile.

During the study period, 240 patients were treated with MT for AIS due to LVO. Of these, 36 patients were excluded because there were no data on HbA1c. The remaining 204 patients with AIS were included in the study. Direct MT was performed in 83 patients (40.3%), while 121 patients (59.3%) received alteplase before MT. The number of patients included in each GAR quartile was as follows: 49 patients (24%) in the first quartile, 52 patients (25.5%) in the second and third quartiles, and 51 patients (25%) in the fourth quartile. These data are summarized in the flow diagram of the study (Figure 1).

The general characteristics of the enrolled subjects, distinguished by GAR quartiles, are presented in Table 1. We did not observe any differences in age or sex between the four groups. Hypertension was the only vascular risk factor that was significantly more common among patients in the highest quartile. In addition, patients in the fourth quartile had higher fasting glucose levels and HbA1c values than those in the other three quartiles. For patients in the first quartile, baseline systolic blood pressure values were significantly higher among subjects in the third quartile. Stroke etiology and neurological impairment at admission did not differ between the four quartiles. At discharge, patients in the fourth quartile showed significantly higher NIHSS scores than those in the other three quartiles. Almost all patients treated with MT in our center had a pre-stroke mRS score of ≤ 2.

**Table 1:** General characteristics of the subjects according to the GAR quartiles.

	**GAR Q1** ** (*n* = 49)**	**GAR Q2** ** (*n* = 52)**	**GAR Q3** ** (*n* = 52)**	**GAR Q4** ** (*n* = 51)**	***p***
**Demographic data**					
Age, years*	74 (67.5–81.5)	73.5 (64–81.5)	75 (67.2–80.7)	75 (69–81)	0.953
Males, *n* (%)	24 (49)	23 (44.2)	29 (55.8)	24 (47.1)	0.681
**Laboratory findings**					
Hb, g/dl	12.4 ± 1.5	12.4 ± 1.5	12.8 ± 1.9	12.9 ± 1.8	0.268
Platelets, 10^3^/mmc*	204 (160.5–255.5)	191 (151.1–233.2)	175 (141.1–225)	189 (151–238)	0.190
aPTT ratio*	0.97 (0.89–1.09)	0.95 (0.87–1.02)	0.94 (0.85–1.04)	0.92 (0.84–1.02)	0.309
INR*	1.1 (1.03–1.17)	1.1 (1.02–1.2)	1.09 (1.03–1.2)	1.1 (1.02–1.22)	0.872
Creatinine, mg/dl*	0.84 (0.74–1.04)	0.85 (0.77–1.04)	0.92 (0.75–1.08)	0.99 (0.78–1.14)	0.321
C-reactive protein, mg/l*	4.83 (2.39–14.49)	8.25 (3.49–14.42)	6.56 (2.78–15.25)	7.41 (3.12–14.39)	0.816
Protein, g/dl*	5.9 (5.5–6.4)	6 (5.7–6.5)	6 (5.6–6.6)	6.1 (5.7–6.6)	0.727
Fasting plasma glucose, mg/dl*	86 (82–90.5)	98.5 (91.5–106)	110.5 (106–121)	156 (138–187)	0.001
HbA1c values, %*	5.8 (5.6–6.1)	5.6 (5.4–5.9)	5.7 (5.2–6.1)	5.9 (5.6–6.5)	0.001
GAR index*	15.1 (13.6–15.7)	17.6 (16.9–18.1)	19.8 (18.9–20.8)	25.4 (23.1–28.4)	0.001
Total cholesterol, mg/dl*	155 (134–180.5)	167 (147–193)	175 (149–191)	162 (139–187.5)	0.210
HDL cholesterol, mg/dl*	47 (38.5–62)	51 (41–61)	51 (45–61)	51 (39–59)	0.379
LDL cholesterol, mg/dl	90.6 ± 30.8	96 ± 29	93.9 ± 34.2	96.4 ± 39.2	0.774
Triglycerides, mg/dl*	87 (63.5–130.5)	85 (64–105)	93 (78.5–131.5)	84 (62.2–143.7)	0.282
**Blood pressure**					
Systolic blood pressure, mmHg	143.2 ± 25.7	150.8 ± 20.8	156.4 ± 20	155.4 ± 20.6	0.031
**Antithrombotic treatment at admission**					
Antiplatelets, *n* (%)	18 (36.7)	10 (19.2)	12 (23.1)	14 (27.5)	0.223
Anticoagulants, *n* (%)	6 (12.2)	10 (19.2)	10 (19.2)	11 (21.6)	0.652
**Antidiabetic drugs at admission**						
Oral hypoglycemics, *n* (%)	3 (6.1)	2 (3.8)	6 (11.5)	5 (9.8)	0.457
Insulin, *n* (%)	0 (0)	0 (0)	0 (0)	1 (2)	0.389
**Vascular risk factors**					
Previous transient ischemic attack/stroke, *n* (%)	7 (14.3)	4 (7.7)	1 (1.9)	8 (15.7)	0.071
Cardiovascular disease, *n* (%)	11 (22.4)	6 (11.5)	6 (11.5)	8 (15.7)	0.378
Atrial fibrillation, *n* (%)	16 (32.7)	14 (26.9)	12 (23.1)	14 (27.5)	0.760
Hypertension, *n* (%)	33 (67.3)	35 (67.3)	33 (63.5)	44 (86.3)	0.048
Diabetes mellitus, *n* (%)	4 (8.2)	2 (3.8)	7 (13.5)	8 (15.7)	0.191
Hypercholesterolemia, *n* (%)	12 (24.5)	12 (23.1)	15 (28.8)	12 (23.5)	0.901
Current smoking, *n* (%)	6 (12.2)	6 (11.5)	9 (17.3)	8 (15.7)	0.811
Median baseline ASPECTS score (range)	10 (8–10)	10 (7–10)	10 (7–10)	10 (7–10)	0.280
**Stroke subtypes based on TOAST classification**					0.691
Large arterial atherosclerosis, *n* (%)	4 (8.2)	9 (17.3)	6 (11.5)	5 (9.8)	
Cardioembolism, *n* (%)	28 (57.1)	27 (51.9)	28 (53.8)	27 (52.9)	
Other determined etiology, *n* (%)	0 (0)	3 (5.8)	2 (3.8)	1 (2)	
Undetermined etiology, *n* (%)	17 (34.7)	13 (25)	16 (30.8)	18 (35.3)	
**Baseline clinical characteristics**					
Median NIHSS score at admission (IQR)	15 (11.5–20)	17 (12–19)	17 (16–20)	17 (14–21)	0.176
Median NIHSS score at discharge (IQR)	2 (0–9)	2 (0.5–11.5)	5 (1–10)	12.5 (3–17)	0.001
Pre-stroke mRS 0–2, *n* (%)	43 (87.8)	50 (96.2)	49 (94.2)	48 (94.1)	0.371

*GAR Q1, first glucose-to-glycated hemoglobin ratio quartile; GAR Q2, second glucose-to-glycated hemoglobin ratio quartile; GAR Q3, third glucose-to-glycated hemoglobin ratio quartile; GAR Q4, fourth glucose-to-glycated hemoglobin ratio quartile; Hb, hemoglobin; aPTT, activated partial thromboplastin time; INR, international normalized ratio; HbA1c, glycated hemoglobin; GAR, glucose-to-glycated hemoglobin ratio; HDL, high-density lipoprotein; LDL, low-density lipoprotein; ASPECTS, Alberta Stroke Program Early CT Score; TOAST, Trial of ORG 10172 in Acute Stroke Treatment; MT, mechanical thrombectomy; NIHSS, National Institutes of Health Stroke Scale; mRS, modified Rankin Scale*.

Data are presented as mean and standard deviation for normally distributed continuous variables. Non-normally distributed continuous variables are displayed as median and interquartile range and are identified by an asterisk (*).

Table 2 summarizes the information on the thrombectomy procedure in the four quartiles. As expected, MCA was the most common site of LVO in all four quartiles. We did not detect any difference in the type of device used for MT and the number of patients receiving alteplase before MT. Similarly, the median time from onset of symptoms and hospital arrival to MT was not significantly different among the four quartiles. A trend toward a decreased prevalence of successful recanalization was observed in patients with more severe stress hyperglycemia.

**Table 2:** Information on thrombectomy procedure according to the GAR quartiles.

	**GAR Q1** ** (*n* = 49)**	**GAR Q2** ** (*n* = 52)**	**GAR Q3** ** (*n* = 52)**	**GAR Q4** ** (*n* = 51)**	***p***
**Site of LVO**					0.425
MCA, *n* (%)	39 (79.6)	43 (82.7)	40 (76.9)	39 (76.5)	
Tandem, *n* (%)	4 (8.2)	8 (15.4)	9 (17.3)	8 (15.7)	
Vertebrobasilar, *n* (%)	6 (12.2)	1 (1.9)	3 (5.8)	4 (7.8)	
**Type of device use for MT**					0.444
Thromboaspiration, *n* (%)	26 (53.1)	23 (44.2)	22 (42.3)	22 (43.1)	
Stent retriever, *n* (%)	2 (4.1)	1 (1.9)	2 (3.8)	5 (9.8)	
Thromboaspiration plus stent retriever, *n* (%)	15 (30.6)	19 (36.5)	24 (46.2)	20 (39.2)	
Permanent stenting, *n* (%)	6 (12.2)	9 (17.3)	4 (7.7)	4 (7.8)	
**Other information on recanalization therapy**					
Alteplase use prior to MT, *n* (%)	31 (63.3)	28 (53.8)	33 (63.5)	29 (56.9)	0.692
Time from onset of symptoms to alteplase, min*	127.5 (97–166.2)	125 (107.5–180)	152.5 (110.2–180)	151.5 (100–166.2)	0.604
Door-to-needle time, min*	53.5 (42–69.7)	50 (40–67.5)	54 (35–73.7)	64 (42.5–86.5)	0.462
Time from onset of symptoms to MT, min*	205 (165–290)	212.5 (186.2–251.2)	210 (146.2–255)	210 (165–265)	0.844
Door-to-groin time, min*	116 (103–165)	118 (86.2–152.7)	112 (75–143.7)	122 (85–155)	0.489
Procedure length, min*	70 (40–94)	62.5 (46.2–95)	60 (46.2–93.7)	70 (45–100)	0.734
Secondary embolization, *n* (%)	1 (2)	5 (9.6)	4 (7.7)	2 (3.9)	0.351
TICI 2b−3 after MT, n (%)	41 (85.4)	43 (91.5)	40 (88.9)	39 (79.6)	0.360

*GAR Q1, first glucose-to-glycated hemoglobin ratio quartile; GAR Q2, second glucose-to-glycated hemoglobin ratio quartile; GAR Q3, third glucose-to-glycated hemoglobin ratio quartile; GAR Q4, fourth glucose-to-glycated hemoglobin ratio quartile; LVO, large vessel occlusion; MCA, middle cerebral artery; MT, mechanical thrombectomy; TICI, Thrombolysis in Cerebral Infarction*.

Data are presented as mean and standard deviation for normally distributed continuous variables. Non-normally distributed continuous variables are displayed as median and interquartile range and are identified by an asterisk (*).

### Association of Stress Hyperglycemia With Clinical Outcomes in Univariate Analysis

**Figure 2:**
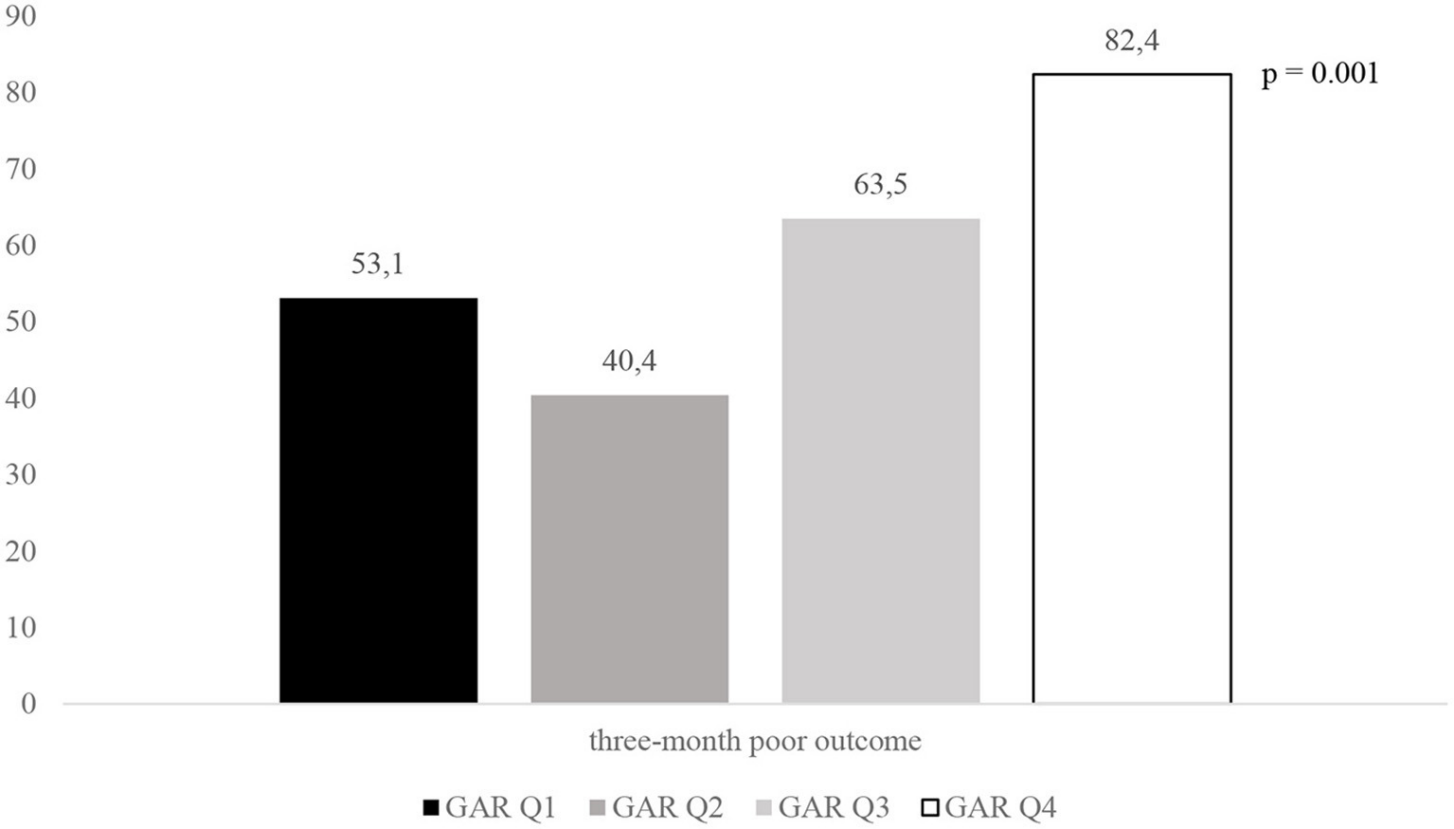
Rates of 3-month poor outcome according to GAR quartile. GAR Q1, first glucose-to-glycated hemoglobin ratio quartile; GAR Q2, second glucose-to-glycated hemoglobin ratio quartile; GAR Q3, third glucose-to-glycated hemoglobin ratio quartile; GAR Q4, fourth glucose-to-glycated hemoglobin ratio quartile.

**Figure 3:**
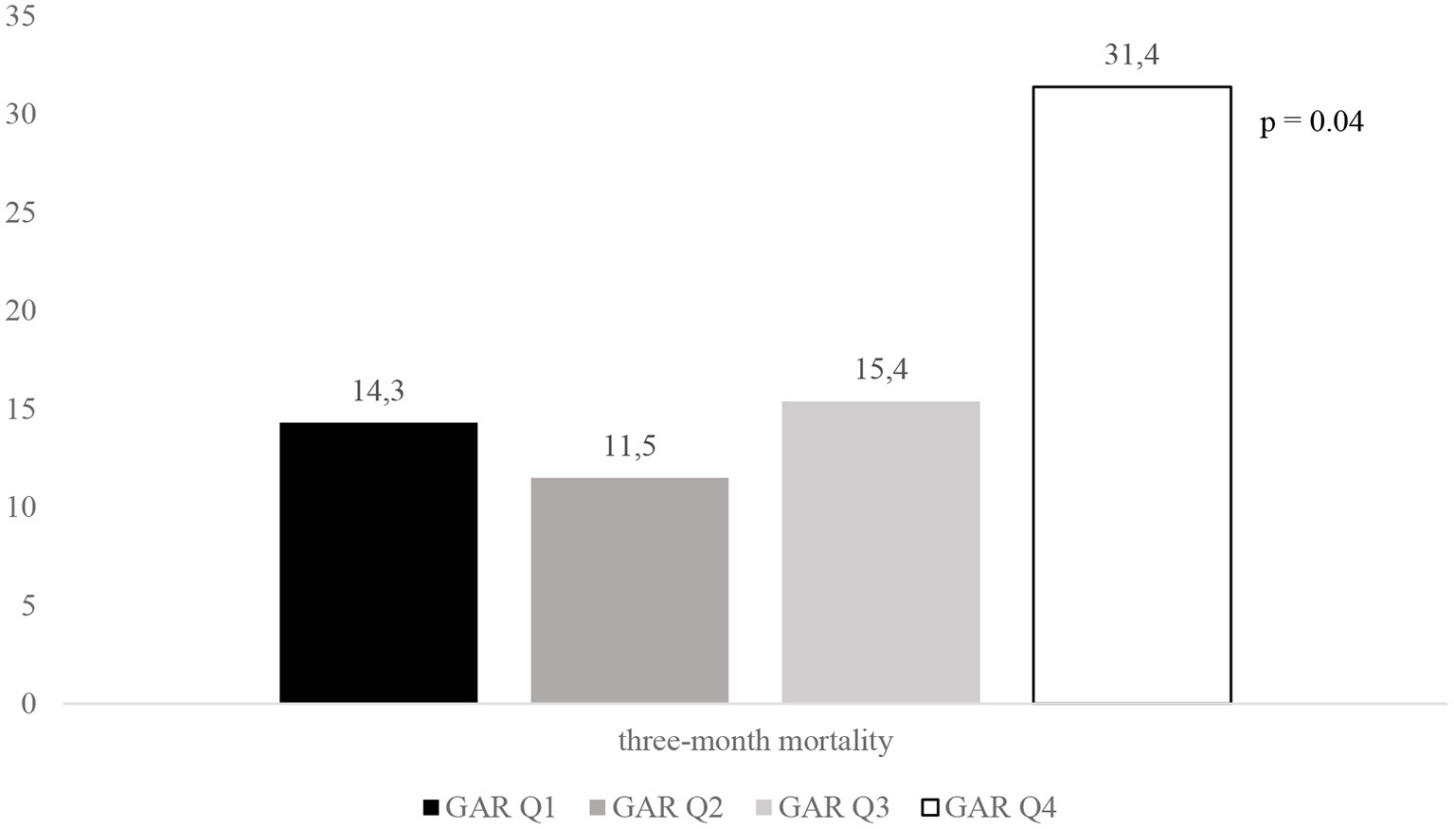
Rates of 3-month mortality according to GAR quartile. GAR Q1, first glucose-to-glycated hemoglobin ratio quartile; GAR Q2, second glucose-to-glycated hemoglobin ratio quartile; GAR Q3, third glucose-to-glycated hemoglobin ratio quartile; GAR Q4, fourth glucose-to-glycated hemoglobin ratio quartile.

**Figure 4:**
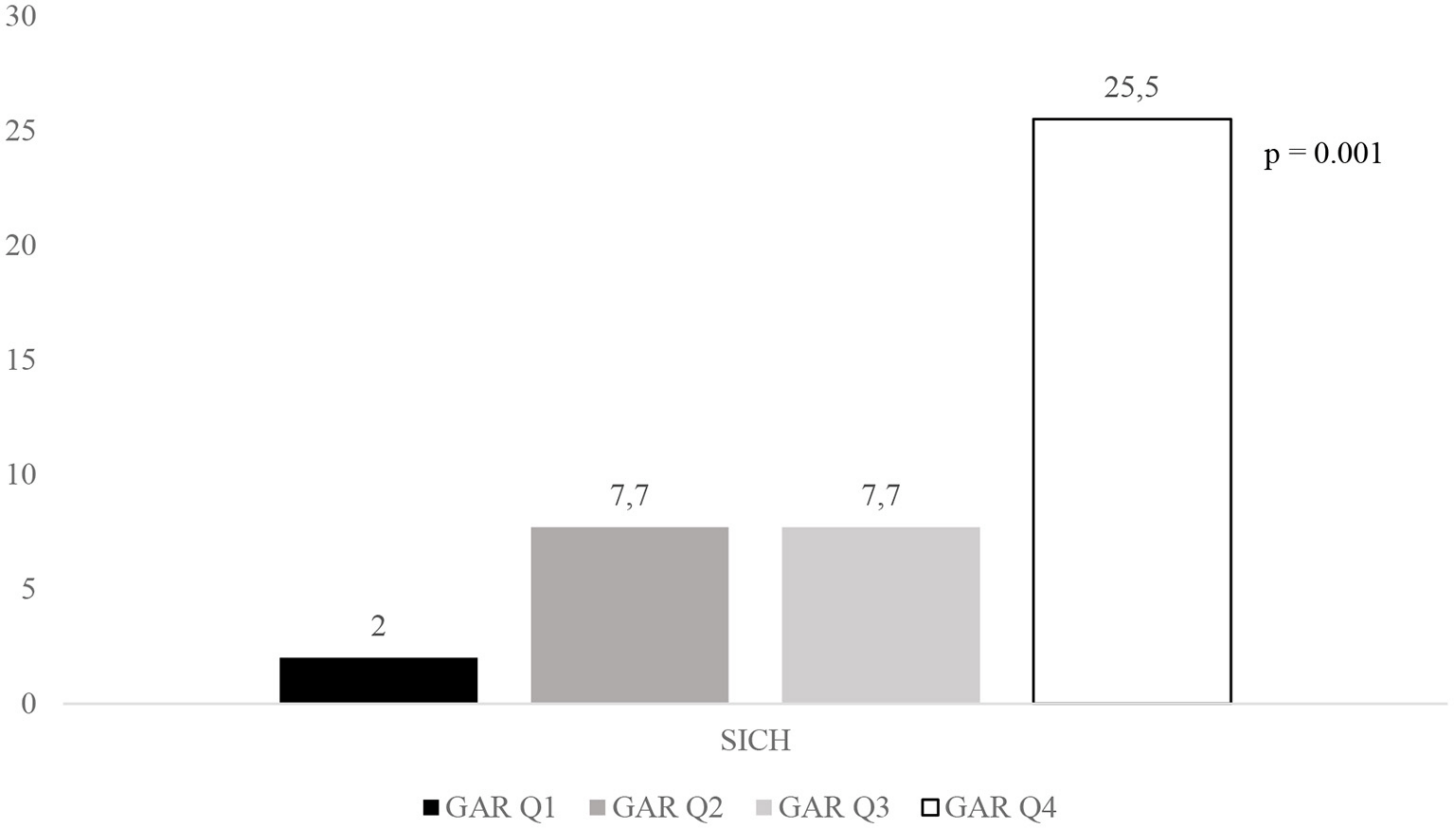
Rates of SICH according to GAR quartile. SICH, symptomatic intracranial hemorrhage; GAR Q1, first glucose-to-glycated hemoglobin ratio quartile; GAR Q2, second glucose-to-glycated hemoglobin ratio quartile; GAR Q3, third glucose-to-glycated hemoglobin ratio quartile; GAR Q4, fourth glucose-to-glycated hemoglobin ratio quartile.

Figures 2–4 report the rates of 3-month poor outcome, 3-month mortality, and SICH according to GAR quartiles. The prevalence of no major neurological improvement (Q1, 23.4%; Q2, 26.5%; Q3, 19.1%; Q4 69.2%; *p* = 0.001), in-hospital mortality (Q1, 4.1%; Q2, 5.8%; Q3, 9.6%; Q4, 21.6%; *p* = 0.02), and ICH (Q1, 8.2%; Q2, 17.3%; Q3, 32.7%; Q4, 45.1%; *p* = 0.001) were significantly different among the four quartiles.

### Association of Stress Hyperglycemia With Clinical Outcomes in Multivariate Analysis

All outcome measures were significantly associated with severe stress hyperglycemia, even after controlling for confounders (Table 3). Independent predictors, other than the highest GAR quartile, were the following: ASPECTS score (OR 0.46, 95% CI 0.24–0.88, *p* = 0.02), NIHSS score at admission (OR 1.18, 95% CI 1.08–1.28, *p* = 0.001), pre-stroke mRS (OR 3.29, 95% CI 1.34–8.07, *p* = 0.009), door-to-groin time (OR 1.01, 95% CI 1.00–1.02, *p* = 0.048), and procedure duration (OR 1.01, 95% CI 1.00–1.02, *p* = 0.035) for 3-month poor outcome; procedure duration (OR 1.02, 95% CI 1.01–1.03, *p* = 0.002) for no major neurological improvement; ASPECTS score (OR 0.56, 95% CI 0.31–0.98, *p* = 0.045) and pre-stroke mRS (OR 1.71, 95% CI 1.19–2.44, *p* = 0.003) for 3-month mortality; NIHSS score at admission (OR 1.1, 95% CI 1.01–1.2, *p* = 0.024) and systolic blood pressure (OR 1.03, 95% CI 1.01–1.05, *p* = 0.01) for ICH. In contrast, in-hospital mortality and SICH were not associated with variables other than the fourth quartile.

**Table 3:** Logistic regression model: adjusted ORs (95% CIs) of the GAR quartiles in relation to the respective outcomes.

	**GAR Q1**	**GAR Q2**	**GAR Q3**	**GAR Q4**
Three-month poor outcome^†^	1	0.43 (0.15–1.22) *p* = 0.114	1.32 (0.47–3.71) *p* = 0.6	4.52 (1.4–14.62) *p* = 0.012
No major neurological improvement at discharge^†^	1	1.46 (0.51–4.23) *p* = 0.481	0.76 (0.24–2.37) *p* = 0.635	9.11 (2.94–28.23) *p* = 0.001
Three-month mortality^†^	1	1.05 (0.27–4.16) *p* = 0.942	1.59 (0.43–5.94) *p* = 0.488	3.55 (1.02–12.29) *p* = 0.046
In-hospital mortality^†^	1	1.52 (0.24–9.67) *p* = 0.658	2.79 (0.49–15.69) *p* = 0.245	6.79 (1.38–33.37) *p* = 0.018
Presence of SICH^‡^	1	3.74 (0.34–40.73) *p* = 0.278	3.86 (0.4–37.2) *p* = 0.242	11.22 (1.27–99.24) *p* = 0.03
Presence of ICH^‡^	1	1.95 (0.46–8.35) *p* = 0.366	2.89 (0.76–11.02) *p* = 0.119	6.89 (1.87–25.36) *p* = 0.004

*GAR Q1, first glucose-to-glycated hemoglobin ratio quartile; GAR Q2, second glucose-to-glycated hemoglobin ratio quartile; GAR Q3, third glucose-to-glycated hemoglobin ratio quartile; GAR Q4, fourth glucose-to-glycated hemoglobin ratio quartile; SICH, symptomatic intracranial hemorrhage; ICH, intracranial hemorrhage; ASPECTS, Alberta Stroke Program Early CT Score; MT, mechanical thrombectomy; NIHSS, National Institutes of Health Stroke Scale; mRS, modified Rankin Scale; OR, odds ratio. Adjusted for age, history of diabetes, ASPECTS, baseline NIHSS score, pre-stroke mRS, time from symptom onset to MT, door-to-groin time, and procedure duration. Adjusted for age, history of diabetes, ASPECTS, baseline NIHSS score, pre-stroke mRS, time from symptom onset to MT, door-to-groin time, procedure duration, and systolic blood pressure*.

## Discussion

We demonstrated that independently of ethnicity, stress hyperglycemia is an independent predictor of worse outcomes not only in Chinese patients but also in Caucasian patients with AIS undergoing MT (21, 22).

The association between glucose level at admission and worse outcomes after MT in AIS patients is well known (14–17). In 2016, Kim et al. (14) reported that patients with hyperglycemia (>140 g/dl) exhibited an impaired outcome more frequently at 3 months than patients without hyperglycemia. These data were later reported by other authors (15–17). Since hyperglycemia in AIS represents a dynamic condition, some studies have investigated the contribution of the dynamic patterns of hyperglycemia to stroke outcomes among patients receiving alteplase. These trials revealed that, in addition to a single glucose measurement, the relative blood glucose changes should be considered in the prediction of stroke outcomes (28–30). This is true not only for AIS patients receiving alteplase but also for those subjects with LVO undergoing MT. Indeed, we previously reported that poor functional outcome, mortality, and hemorrhagic transformation after endovascular treatment were significantly associated with the presence of persistent hyperglycemia—that is, hyperglycemia at baseline *plus* at 24 h (18). Although our study provided new insights into the role of impaired glucose metabolism as a predictor of outcome in AIS patients undergoing MT, we were not able to discern between the detrimental effects due to the presence of underlying diabetes and those associated with stress hyperglycemia.

While diabetes is a recognized risk factor for cerebrovascular diseases (19), the consequences of stress hyperglycemia are not well-established. The latter is usually defined as spontaneously resolving hyperglycemia after acute illness dissipation (31). Stress hyperglycemia is mediated by the hypothalamic–pituitary–adrenal axis, the sympatho-adrenal system, and pro-inflammatory cytokines that cause a stress response with excessive gluconeogenesis, glycogenolysis, and insulin resistance (32). Although several studies have reported that stress hyperglycemia increases the risk of poor outcomes in AIS patients (33–36), very few studies have investigated the impact of stress hyperglycemia in stroke patients treated with recanalization therapy (20–22).

The GAR index has been used to quantify stress hyperglycemia. A study by Su et al. (23), which enrolled patients with plasma glucose concentrations >500 mg/dl, reported that GAR independently predicted 90-day mortality, intensive care unit (ICU) admission, and use of mechanical ventilation. Another study showed that the odds of stroke recurrence and all-cause death were significantly increased in non-diabetic patients affected by stress hyperglycemia, as estimated by the GAR index (37). Recently, we demonstrated an independent association between stress hyperglycemia and impaired clinical outcomes in AIS patients undergoing alteplase (20).

To date, only two Chinese studies have investigated the role of stress hyperglycemia as a predictor of poor outcomes in AIS patients with LVO undergoing MT (21, 22). In particular, Wang et al. (21) included 321 patients with ischemic stroke who were treated with MT. They reported that the incidence of 3-month mortality was significantly higher in patients affected by stress hyperglycemia than in those with normoglycemia (21). In a smaller study by Chen et al. (22), which included 160 consecutive AIS patients treated with MT, increased values of stress hyperglycemia represented an independent predictor of poor outcome, defined as a 3-month mRS score of 3–6, also after controlling for multiple potential confounders. In our sample of Caucasian patients, the presence of stress hyperglycemia was independently associated with all outcome measures. In particular, despite the endovascular treatment, a very large proportion of patients with more severe stress hyperglycemia, that is, more than 80% of them, were functionally dependent/dead 3 months after the stroke, and they showed a mortality rate as high as 31.4% at follow-up, and more than a quarter of them were affected by symptomatic hemorrhagic transformation. The detrimental effect of stress hyperglycemia was also confirmed after controlling for all the other variables that could impair outcomes in AIS patients treated with recanalization therapy.

The mechanisms underlying the association between stress hyperglycemia and poor clinical outcomes in stroke patients undergoing MT are incompletely understood. Several hypotheses have been proposed: (1) hyperglycemia might directly cause toxic damage to the ischemic brain due to accumulation of lactate and intracellular acidosis (38); (2) stress-induced inflammatory response may increase circulating free fatty acids in patients with acute illness, thus impairing endothelium-dependent vasodilation (39) and promoting intracellular calcium overload (40); and (3) stress hyperglycemia may lead to reperfusion injury due to increased oxidative stress and inflammation (41).

Our results suggest that stress hyperglycemia should be promptly diagnosed and carefully treated in AIS patients with LVO undergoing MT. However, there is no universally accepted insulin regimen for glycemic control in critically ill patients. In these subjects, the aim of the treatment should be to limit fluctuations in blood glucose levels. The ideal protocol should quickly achieve and maintain the target blood glucose levels to prevent hyperglycemia but also lead to a minimal incidence of hypoglycemia. The SHINE trial, randomizing AIS patients to receive continuous intravenous insulin (intensive treatment group) or subcutaneous insulin on a sliding scale (standard treatment group), reported similar rates of favorable outcomes between the two groups. However, severe hypoglycemic events occurred more frequently in the intensive treatment group (42). The reason why the effects of stress hyperglycemia on the outcome are not influenced by the type of treatment, whether aggressive or standard insulin treatment, remains a puzzle. It is possible that, in the aggressive treatment, the greater benefit of damage produced by hyperglycemia is counterbalanced by the presence of more episodes of hypoglycemia. On the contrary, the standard insulin treatment, although obtaining the correction of hyperglycemia in a longer time interval, is likely less capable of causing hypoglycemic peaks.

Our patients undergoing MT were significantly older than the ones enrolled in previous randomized trials on MT for AIS (43). It probably reflects the fact that the population of Italy is getting older every year, becoming the oldest population in the world. This is particularly true for the Friuli Venezia Giulia, where the city of Udine is located, that represents one of the *oldest* regions in Italy. Although old age is associated with higher mortality and increased disability after AIS (44), we are confident that this risk factor did not affect our results on stress hyperglycemia and poor outcome. In fact, the median age did not differ between the four groups and, moreover, the multivariate analysis was controlled including age as a possible confounding factor.

Our study had several limitations. Since this was a retrospective observational study, the cause–effect relationship between stress hyperglycemia and outcome should be considered speculative. The retrospective nature of the study and the quartile-based analysis might have affected the adequate control for confounding variables. Finally, the relatively small sample size may have limited the statistical power; thus, differences between patients with mild-to-moderate hyperglycemia and those normoglycemic could not be detected.

In conclusion, stress hyperglycemia, as measured by the GAR index, seems to be associated with worse outcomes in AIS patients undergoing MT. In particular, the odds of disability, mortality, and hemorrhagic complications were significantly increased in patients with more severe stress hyperglycemia than in those with normoglycemia. Further studies with larger sample sizes are needed to confirm these preliminary results.

## Data Availability Statement

The raw data supporting the conclusions of this article will be made available by the authors, without undue reservation.

## Ethics Statement

The studies involving human participants were reviewed and approved by Comitato Etico Unico Regionale (Ref. No. CEUR-2020-Os-173). The patients/participants provided their written informed consent to participate in this study.

## Author Contributions

GM and SP contributed to conceptualization and methodology. SP and SL contributed software. SL, GG, and MV contributed to validation. SP, SL, MS, CS, and AS contributed to the investigation. SP, SL, CS, and AS contributed resources. GM contributed to formal analysis and data curation, writing—original draft preparation, and writing—review and editing. GG contributed to visualization. MV contributed to supervision.

## Conflict of Interest

The authors declare that the research was conducted in the absence of any commercial or financial relationships that could be construed as a potential conflict of interest.

## Publisher's Note

All claims expressed in this article are solely those of the authors and do not necessarily represent those of their affiliated organizations, or those of the publisher, the editors and the reviewers. Any product that may be evaluated in this article, or claim that may be made by its manufacturer, is not guaranteed or endorsed by the publisher.

## References

[1] Powers WJ Rabinstein AA Ackerson T Adeoye OM Bambakidis NC Becker K. Guidelines for the early management of patients with acute ischemic stroke: 2019 update to the 2018 guidelines for the early management of acute ischemic stroke: a guideline for healthcare professionals from the American Heart Association/American Stroke Association. Stroke. (2019) 50:e344–418. 10.1161/STR.0000000000000211 31662037

[2] Campbell BC Mitchell PJ Kleinig TJ Dewey HM Churilov L Yassi N. Endovascular therapy for ischemic stroke with perfusion-imaging selection. N Engl J Med. (2015) 372:1009–18. 10.1056/NEJMoa1414792 25671797

[3] Berkhemer OA Fransen PS Beumer D van der Berg LA Lingsma HF Yoo AJ. A randomized trial of intraarterial treatment for acute ischemic stroke. N Engl J Med. (2015) 372:11–20. 10.1056/NEJMoa1411587 25517348

[4] Goyal M Demchuk AM Menon BK Eesa M Rempel JL Thornton J. Randomized assessment of rapid endovascular treatment of ischemic stroke. N Engl J Med. (2015) 372:1019–30. 10.1056/NEJMoa1414905 25671798

[5] Saver JL Goyal M Bonafe A Diener H Levy EI Pereira VM. Stent-retriever thrombectomy after intravenous t-PA vs. t-PA alone in stroke. N Engl J Med. (2015) 372:2285–95. 10.1056/NEJMoa1415061 25882376

[6] Jovin TG Chamorro A Cobo E de Miquel MA Molina CA Rovira A. Thrombectomy within 8 h after symptom onset in ischemic stroke. N Engl J Med. (2015) 372:2296–306. 10.1056/NEJMoa1503780 25882510

[7] Merlino G Sponza M Petralia B Vit A Gavrilovic V Pellegrin A. Short and long-term outcomes after combined intravenous thrombolysis and mechanical thrombectomy versus direct mechanical thrombectomy: a prospective single-center study. J Thromb Thrombolysis. (2017) 44:203–9. 10.1007/s11239-017-1527-8 28702769

[8] Sallustio F Koch G Alemseged F Konda D Fabiano S Pampana E. Effect of mechanical thrombectomy alone or in combination with intravenous thrombolysis for acute ischemic stroke. J Neurol. (2018) 265:2875–80. 10.1007/s00415-018-9073-7 30276519

[9] Minnerup J Wersching H Teuber A Wellmann J Eyding J Weber R. Outcome after thrombectomy and intravenous thrombolysis in patients with acute ischemic stroke: a prospective observational study. Stroke. (2016) 47:1584–92. 10.1161/STROKEAHA.116.012619 27217508

[10] Rom S Zuluaga-Ramirez V Gajghate S Seliga A Winfield M Heldt NA. Hyperglycemia-driven neuroinflammation compromises BBB leading to memory loss in both diabetes mellitus (DM) type 1 and type 2 mouse models. Mol Neurobiol. (2019) 56:1883–96. 10.1007/s12035-018-1195-5 29974394 PMC6320739

[11] Desilles JP Syvannarath V Ollivier V Journé C Delbosc S Ducroux C. Exacerbation of thromboinflammation by hyperglycemia precipitates cerebral infarct growth and hemorrhagic transformation. Stroke. (2017) 48:1932–40. 10.1161/STROKEAHA.117.017080 28526762

[12] Robbins NM Swanson RA. Opposing effects of glucose on stroke and reperfusion injury: acidosis, oxidative stress, and energy metabolism. Stroke. (2014) 45:1881–86. 10.1161/STROKEAHA.114.004889 24743441 PMC4102697

[13] Won SJ Tang XN Suh SW Yenari MA Swanson RA. Hyperglycemia promotes tissue plasminogen activator-induced haemorrhage by Increasing superoxide production. Ann Neurol. (2011) 70:583–90. 10.1002/ana.22538 22002675 PMC4554391

[14] Kim JT Jahan R Saver JL; SWIFT Investigators. Impact of glucose on outcomes in patients treated with mechanical thrombectomy: a *post hoc* analysis of the solitaire flow restoration with the intention for thrombectomy study. Stroke. (2016) 47:120–7. 10.1161/STROKEAHA.115.010753 26658447

[15] Goyal N Tsivgoulis G Pandhi A Dillard K Katsanos AH Magoufis G. Admission hyperglycemia and outcomes in large vessel occlusion strokes treated with mechanical thrombectomy. J Neurointerv Surg. (2018) 10:112–7. 10.1136/neurintsurg-2017-012993 28289148

[16] Osei E den Hertog HM Berkhemer OA Fransen PSS Roos YBWEM Beumer D. Admission glucose and effect of intra-arterial treatment in patients with acute ischemic stroke. Stroke. (2017) 48:1299–305. 10.1161/STROKEAHA.116.016071 28389610

[17] Chamorro Á Brown S Amaro S Hill MD Muir KW Dippel DWJ. Glucose modifies the effect of endovascular thrombectomy in patients with acute stroke. Stroke. (2019) 50:690–6. 10.1161/STROKEAHA.118.023769 30777000

[18] Merlino G Smeralda C Sponza M Gigli GL Lorenzut S Marini A. Dynamic hyperglycemic patterns predict adverse outcomes in patients with acute ischemic stroke undergoing mechanical thrombectomy. J Clin Med. (2020) 9:1932. 10.3390/jcm9061932 32575739 PMC7355777

[19] Chen R Ovbiagele B Feng W. Diabetes and Stroke: epidemiology, pathophysiology, pharmaceuticals and outcomes. Am J Med Sci. (2016) 351:380–6. 10.1016/j.amjms.2016.01.011 27079344 PMC5298897

[20] Merlino G Smeralda C Gigli GL Lorenzut S Pez S Surcinelli A. Stress hyperglycemia is predictive of worse outcome in patients with acute ischemic stroke undergoing intravenous thrombolysis. J Thromb Thrombolysis. (2021) 51:789–97. 10.1007/s11239-020-02252-y 32830310

[21] Wang L Zhou Z Tian X Wang H Yang D Hao Y. ACTUAL investigators. Impact of relative blood glucose changes on mortality risk of patient with acute ischemic stroke and treated with mechanical thrombectomy. J Stroke Cerebrovasc Dis. (2019) 28:213–9. 10.1016/j.jstrokecerebrovasdis.2018.09.036 30539756

[22] Chen X Liu Z Miao J Zheng W Yang Q Ye X. High stress hyperglycemia ratio predicts poor outcome after mechanical thrombectomy for ischemic stroke. J Stroke Cerebrovasc Dis. (2019) 28:1668–73. 10.1016/j.jstrokecerebrovasdis.2019.02.022 30890395

[23] Su YW Hsu CY Guo YW Chen HS. Usefulness of the plasma glucose concentration-to-HbA1c ratio in predicting clinical outcomes during acute illness with extreme hyperglycaemia. Diabetes Metab. (2017) 43:40–7. 10.1016/j.diabet.2016.07.036 27663631

[24] Pexman JH Barber PA Hill MD Sevick RJ Demchuk AM Hudon ME. Use of the Alberta stroke program early CT score (ASPECTS) for assessing CT scans in patients with acute stroke. AJNR Am J Neuroradiol. (2001) 22:1534–42. 11559501 PMC7974585

[25] Adams HP Jr Bendixen BH Kappelle LJ Biller J Love BB Gordon DL. Classification of subtype of acute ischemic stroke. Definitions for use in a multicenter clinical trial. TOAST. Trial of Org 10172 in Acute Stroke Treatment. Stroke. (1993) 24:35–41. 10.1161/01.STR.24.1.35 7678184

[26] Hacke W Kaste M Fieschi C Toni D Lesaffre E von Kummer R. Intravenous thrombolysis with recombinant tissue plasminogen activator for acute hemispheric stroke. The European Cooperative Acute Stroke Study (ECASS). JAMA. (1995) 274:1017–25. 10.1001/jama.1995.03530130023023 7563451

[27] Hacke W Kaste M Bluhmki E Brozman M Dávalos A Guidetti D. Thrombolysis with alteplase 3 to 4.5 hours after acute ischemic stroke. N Engl J Med. (2008) 359:1317–29. 10.1056/NEJMoa0804656 18815396

[28] Yong M Kaste M. Dynamic of hyperglycemia as a predictor of stroke outcome in the ECASS-II trial. Stroke. (2008) 39: 2749–55. 10.1161/STROKEAHA.108.514307 18703813

[29] Putaala J Sairanen T Meretoja A Lindsberg PJ Tiainen M Liebkind R. Post-thrombolytic hyperglycemia and 3-month outcome in acute ischemic stroke. Cerebrovasc Dis. (2011) 31:83–92. 10.1159/000321332 21079397

[30] Yoo DS Chang J Kim JT Choi MJ Choi J Choi KH. Various blood glucose parameters that indicate hyperglycemia after intravenous thrombolysis in acute ischemic stroke could predict worse outcome. PLoS ONE. (2014) 9:e94364. 10.1371/journal.pone.0094364 24747428 PMC3991642

[31] Bernard C. Lecons sur les Phenomenes de la Vie Communs aux Animaux et aux Vegetaux. JB Bailliere et fi ls. Paris: Librairie JB Bailliere et fils. (1878). 10.5962/bhl.title.44802

[32] Marik PE Bellomo R. Stress hyperglycemia: an essential survival response! Crit Care Med. (2013) 41:e93–4. 10.1097/CCM.0b013e318283d124 23685597

[33] Badawi O Waite MD Fuhrman SA Zuckerman IH. Association between intensive care unit-acquired dysglycemia and inhospital mortality. Crit Care Med. (2012) 40:3180–8. 10.1097/CCM.0b013e3182656ae5 22971590

[34] Bruno A Levine SR Frankel MR Brott TG Lin Y Tilley BC. Admission glucose level and clinical outcomes in the NINDS rt-PA stroke trial. Neurology. (2002) 59:669–74. 10.1212/WNL.59.5.669 12221155

[35] Capes SE Hunt D Malmberg K Gerstein HC. Stress hyperglycaemia and increased risk of death after myocardial infarction in patients with and without diabetes: a systematic overview. Lancet. (2000) 355:773–8. 10.1016/S0140-6736(99)08415-9 10711923

[36] Dungan K Braithwaite SS Preiser JC. Stress hyperglycemia. Lancet. (2009) 373:1798–807. 10.1016/S0140-6736(09)60553-5 19465235 PMC3144755

[37] Zhu B Pan Y Jing J Meng X Zhao X Liu L. Stress hyperglycemia and outcome of non-diabetic patients after acute ischemic stroke. Front Neurol. (2019) 10:1003. 10.3389/fneur.2019.01003 31620074 PMC6759951

[38] Levine SR Welch KM Helpern JA Chopp M Bruce R Selwa J. Prolonged deterioration of ischemic brain energy metabolism and acidosis associated with hyperglycemia: human cerebral infarction studied by serial 31P NMR spectroscopy. Ann Neurol. (1988) 23:416–8. 10.1002/ana.410230423 3382181

[39] Steinberg HO Tarshoby M Monestel R Hook G Cronin J Johnson A. Elevated circulating free fatty acid levels impair endothelium-dependent vasodilation. J Clin Invest. (1997) 100:1230–9. 10.1172/JCI119636 9276741 PMC508300

[40] Oliver MF Opie LH. Effects of glucose and fatty acids on myocardial ischaemia and arrhythmias. Lancet. (1994) 343:155–8. 10.1016/S0140-6736(94)90939-3 7904009

[41] Luitse M Biessels G Rutten G Kappelle LJ. Diabetes, hyperglycaemia, and acute ischaemic stroke. Lancet Neurol. (2012)11:261–71. 10.1016/S1474-4422(12)70005-4 22341034

[42] Johnston KC Bruno A Pauls Q Hall CE Barrett KM Barsan W. Intensive vs standard treatment of hyperglycemia and functional outcome in patients with acute ischemic stroke: the SHINE randomized clinical trial. JAMA. (2019) 322:326–35. 10.1001/jama.2019.9346 31334795 PMC6652154

[43] Elgendy IY Kumbhani DJ Mahmoud A Bhatt DL Bavry AA. Mechanical thrombectomy for acute ischemic stroke: a meta-analysis of randomized trials. J Am Coll Cardiol. (2015) 66:2498–505. 10.1016/j.jacc.2015.09.070 26653623

[44] Rothwell PM Coull AJ Silver LE Fairhead JF Giles MF Lovelock CE. Population-based study of event-rate, incidence, case fatality, and mortality for all acute vascular events in all arterial territories (Oxford Vascular Study). Lancet. (2005) 366:1773–83. 10.1016/S0140-6736(05)67702-1 16298214

